# Role of Pial Microvasospasms and Leukocyte Plugging for Parenchymal Perfusion after Subarachnoid Hemorrhage Assessed by In Vivo Multi-Photon Microscopy

**DOI:** 10.3390/ijms22168444

**Published:** 2021-08-06

**Authors:** Julian Schwarting, Kathrin Nehrkorn, Hanhan Liu, Nikolaus Plesnila, Nicole Angela Terpolilli

**Affiliations:** 1 Institute for Stroke and Dementia Research, Munich University Hospital, Graduate School of Systemic Neurosciences, Munich Cluster for Systems Neurology (SyNergy), Ludwig-Maximilians-University, 81377 Munich, Germany; julian.schwarting@med.uni-muenchen.de (J.S.); kathrin.nehrkorn@fau.de (K.N.); echo89liu@outlook.com (H.L.); nicole.terpolilli@med.uni-muenchen.de (N.A.T.); 2 Department of Neurosurgery, Munich University Hospital, 81377 Munich, Germany

**Keywords:** subarachnoid hemorrhage, microvasospasm, leukocytes, multi-photon microscopy

## Abstract

Subarachnoid hemorrhage (SAH) is associated with acute and delayed cerebral ischemia. We suggested spasms of pial arterioles as a possible mechanism; however, it remained unclear whether and how pial microvasospasms (MVSs) induce cerebral ischemia. Therefore, we used in vivo deep tissue imaging by two-photon microscopy to investigate MVSs together with the intraparenchymal microcirculation in a clinically relevant murine SAH model. Male C57BL/6 mice received a cranial window. Cerebral vessels and leukocytes were labelled with fluorescent dyes and imaged by in vivo two-photon microscopy before and three hours after SAH induced by filament perforation. After SAH, a large clot formed around the perforation site at the skull base, and blood distributed along the perivascular space of the middle cerebral artery up to the cerebral cortex. Comparing the cerebral microvasculature before and after SAH, we identified three different patterns of constrictions: pearl string, global, and bottleneck. At the same time, the volume of perfused intraparenchymal vessels and blood flow velocity in individual arterioles were significantly reduced by more than 60%. Plugging of capillaries by leukocytes was observed but infrequent. The current study demonstrates that perivascular blood is associated with spasms of pial arterioles and that these spasms result in a significant reduction in cortical perfusion after SAH. Thus, the pial microvasospasm seems to be an important mechanism by which blood in the subarachnoid space triggers cerebral ischemia after SAH. Identifying the mechanisms of pial vasospasm may therefore result in novel therapeutic options for SAH patients.

## 1. Introduction

Microcirculatory dysfunction is a hallmark of early brain injury (EBI) after subarachnoid hemorrhage (SAH) [1,2,3,4,5,6]. Constrictions of pial arterioles, or microvasospasms (MVSs), have been detected in SAH patients [7,8] as well as in animal models of SAH [9,10,11] and are thought to significantly contribute to post-hemorrhagic ischemia. However, it is unclear whether spasms of pial arterioles indeed result in a reduction in parenchymal perfusion. Further, the mechanisms causing MVS formation are far from clear. Nitric oxide (NO) depletion has been identified as a significant contributor of MVS formation and microcirculatory disturbance [12,13,14], as well as microvascular thrombosis [9,15,16] and inflammatory changes [6,17]. Adhesion of inflammatory leukocytes occurs early after SAH in superficial brain vessels [11] and may cause accumulation of neutrophils in brain parenchyma within minutes of SAH [17,18,19]. Furthermore, increased leukocyte counts in cerebrospinal fluid and blood have been shown to correlate with secondary complications and poor overall outcome after SAH [20,21]. It is, however, unclear whether leukocytes contribute to perfusion deficits after SAH, e.g., by plugging of constricted capillaries. The aim of this study was therefore to investigate whether spams of the pial microcirculation cause a reduction in microvascular perfusion within the brain parenchyma and to identify whether leukocytes are involved in this process.

## 2. Results

**Figure 1:**
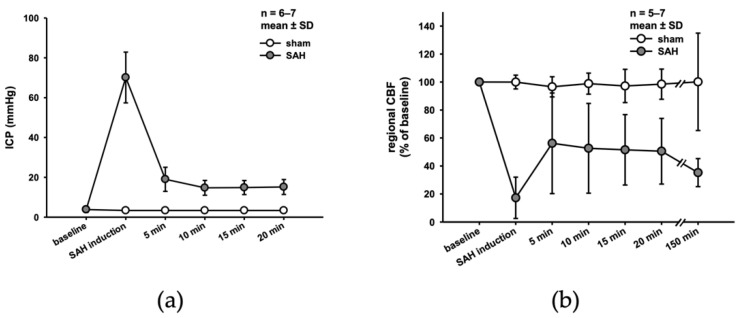
SAH induction. Successful SAH induction is characterized by sharp increase in ICP (**a**), and a significant drop of CBF (**b**).

Systemic blood pressure, core body temperature, heart rate, and oxygen saturation (Supplementary Table S1) as well as respiration parameters (pO_2_, pCO_2_, and pH) and electrolytes (Supplementary Table S2) did not differ between the SAH and sham surgery groups and were within physiological range at the end of the experiment. After SAH induction, ICP values sharply increased to approximately 70 mmHg; within the first few minutes, values dropped and stabilized at around 15 mmHg until the end of the observation period (Figure 1a). At the same time, cerebral blood flow dropped to below 20% of baseline. As with ICP, CBF recovered over time but remained reduced to 65% of baseline up to 150 min after SAH (Figure 1b).

**Figure 2:**
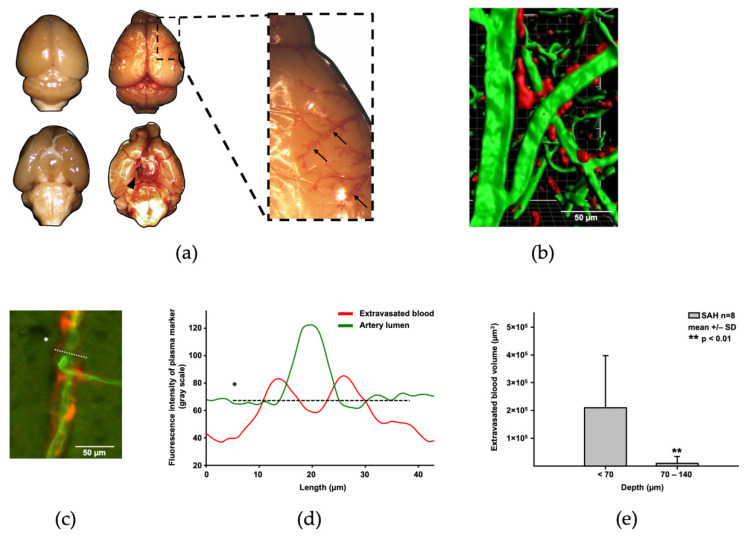
Distribution of subarachnoid blood after SAH. (**a**) Exemplary mouse brain after sham surgery (left) or SAH (right) after washing out all intravascular blood by transcardial perfusion with saline. Blood pools at the perforation site at the skull base (black arrowhead) and distributes along the perivascular space along the curvature of the hemisphere (see the right inlet at higher magnification) creating a railroad track-like pattern. (**b**) This pattern can be confirmed in in vivo microscopy. Blood extravasated during SAH (labelled red with TMRM) can be detected predominantly in the perivascular space, while FITC (green) injected after SAH labels the intravascular plasma. Distribution of both signals can be quantified by measurement of fluorescence intensity of both plasma markers in an axial plane through the vessel (**c**), which confirms the pattern (**d**). Extravasated blood is predominantly found in superficial arterioles (depth less than 70 µm, (**e**)). * Line of interest for measurements of vessel fluorescence intensity.

After SAH induction, subarachnoid blood distributed within the perivascular spaces from the perforation site at the skull base towards the outer curvature of both hemispheres (Figure 2a); after transcardial perfusion (i.e., after washing out all intravascular blood), the perivascular extravasated blood created a “railroad track” pattern along pial vessels (Figure 2a, right side). When blood is labelled with TMRM (red fluorescence) before SAH induction, the perivascular distribution can be differentiated from intravascular blood (labelled with FITC, green fluorescence) and visualized in vivo (Figure 2b). Intensity analysis of the cross-section of a blood-covered blood vessel after SAH (Figure 2c) demonstrated the perivascular distribution of the blood (Figure 2d). Most of the extravasated blood was distributed along pial and superficial cortical vessels (depth > 70 µm) with only little accumulation around deeper vessels, consistent with the increasingly narrower perivascular space around penetrating vessels (Figure 2e). 

**Figure 3:**
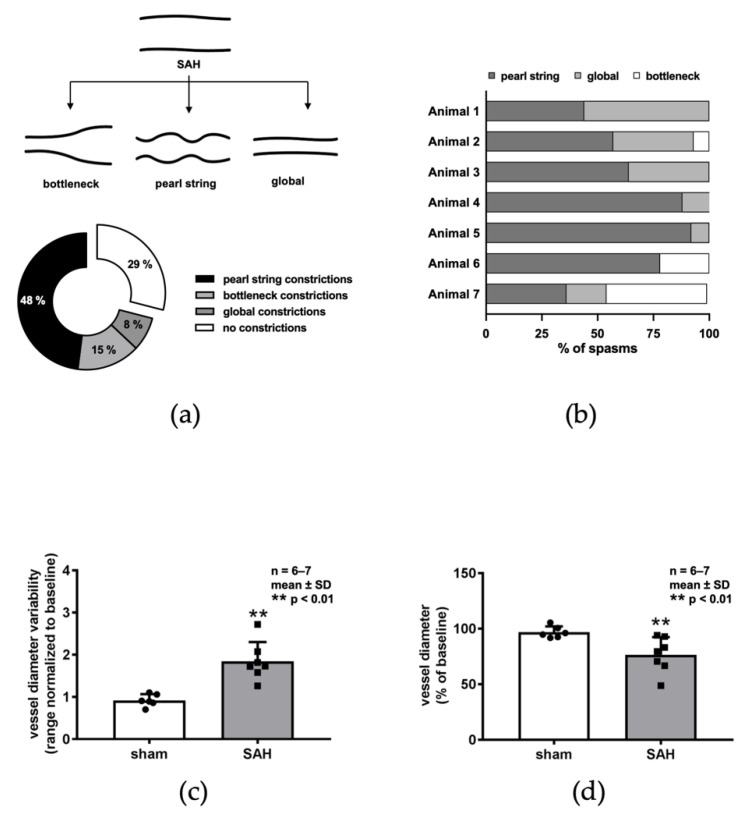
Microvasospasm patterns after SAH. (**a**) Significant microarteriolar constriction can be observed in three different constriction patterns: bottleneck constrictions (1), pearl-string constrictions (2), and global constrictions (3), which are illustrated in (**b**). Distribution between the types varies inter-individually (**c**), with pearl-string shapes being most common. (**d**) Vessel diameter variability, a surrogate parameter for the degree of microvascular abnormalities, was almost doubled compared to sham surgery.

Imaging of the pial vasculature before and after SAH revealed significant microarteriolar constriction of varying morphology. The majority of observed vessel segments showed evidence of the previously described pearl-string-like constrictions (Figure 3a) [9]. However, when compared to baseline, some vessels exhibited alternating constriction patterns: approximately 15% of vessels displayed a uniformly narrowed vessel diameter over longer segments of length, without additional focal constrictions, which we termed “global constriction” (Figure 3a); 8% of spasms were of a “bottleneck” pattern characterized by partial massive constriction ending in an almost unchanged vessel diameter (Figure 3a). Distribution between these types of constriction varied little between individual mice (Figure 3b). To further characterize these microvascular abnormalities, we developed a microvessel variability index that assessed the range of vessel diameters within one vessel segment normalized to baseline diameter. The variability of vessel diameter within segments after SAH was double that of sham animals, indicating significant post-hemorrhagic microvascular diameter alteration (Figure 3c). Overall, the mean cerebral arterial diameter was reduced by 23% after SAH compared to baseline, while sham surgery had negligible effects on the diameter (Figure 3d). 

**Figure 4:**
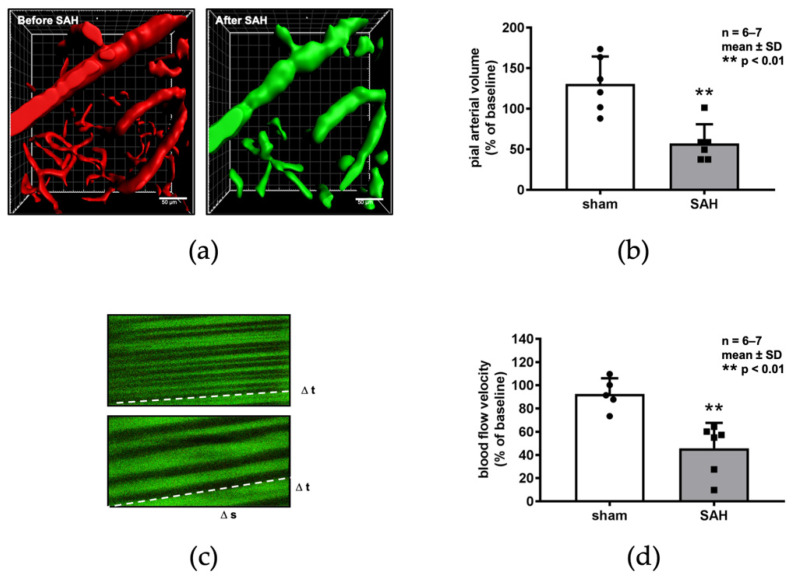
Microcirculatory dysfunction after SAH. Comparison of the cerebral microcirculation by in vivo 2-PM using plasma markers TMRM before ((**a**), left image) and FITC after SAH ((**a**), right image) shows a reduction in the perfused pial artery volume, amounting to 35% of baseline (**b**). Blood flow velocity was measured by linescans as shown in (**c**) and decreased to 54% of baseline as demonstrated in (**d**).

This reduction in vessel diameters of pial arteries translated into a reduction in total perfused vessel volume, a surrogate parameter of microvascular perfusion. Vessel volume was quantified before and after SAH (Figure 4a). Total volume of perfused microvessels was significantly reduced in pial as well as parenchymal arterioles by more than 60% (*p* < 0.01; Figure 4b) after SAH. Focal vasoconstriction could theoretically be compensated by an increase in blood flow velocity to maintain red blood cell delivery to the tissue. However, blood flow velocity measured by line scan analysis (Figure 4c) was significantly reduced by 55% as compared to baseline conditions (*p* < 0.01; Figure 4d). 

**Figure 5:**
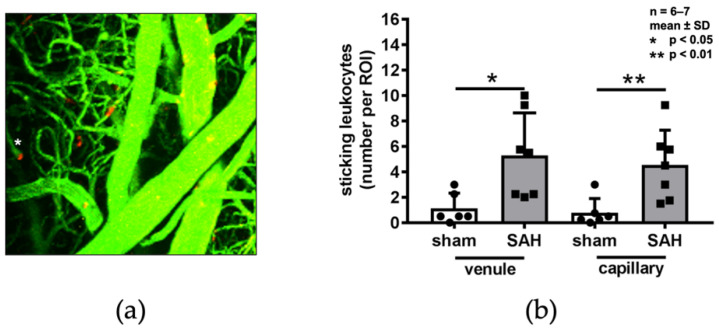
(**a**) Exemplary 3D reconstructions of red fluorescent leucocytes within the vessels stained with FITC (green fluorescence). (**b**). The total amount of leukocytes in venules and capillaries of varying diameters (50–250 µm) was increased compared to that of sham-operated controls; however, the absolute number of leukocytes was low.

Leukocyte–endothelium interactions were assessed in vivo by intravital microscopy 3 h after SAH. Leucocytes that did not change their position between repeated scans of the same regions of interest were quantified as “sticking” (Figure 5a). The number of sticking leukocytes in superficial venules and capillaries increased five-fold after SAH. Nevertheless, their overall number remained small, with an average of six sticking leukocytes detected in venules and capillaries of each ROI (Figure 5b).

## 3. Discussion

In the present study, we describe changes of the cerebral microcirculation in the early phase after SAH by in-vivo two-photon microscopy. In direct comparison of pre- versus post-hemorrhagic imaging in the same animal, pial and parenchymal vessels up to 400 µm in depth constrict in different patterns, leading to a reduction in mean vessel diameter and, ultimately, an impairment of tissue perfusion. Spasms were static, as also previously shown by video microscopy (LIT), and could therefore be differentiated from vasomotion, a dynamic process occurring under conditions of reduced cerebral blood flow.

The closed skull imaging technique used in this study allows observation of the same vessel before and after hemorrhage. This technical improvement led to the de novo identification of different vasoconstriction patterns. Previous experimental investigations by us [9] and others [10,11] assessed microvasospasm formation only after SAH. Hence, the assessment of uniform narrowing of longer or complete vessel segments was not possible due to lack of baseline measurements. Previous studies have therefore underestimated the number and, thus, the significance of MVSs after SAH.

Using line scanning by two-photon microscopy, we were also able for the first time to measure blood flow velocity in the cerebral microcirculation before and after SAH. Blood flow velocity was found to be reduced by more than half (54%) compared to baseline, indicating significant microcirculatory deficit, in line with observations in patients [2,3].

The interaction of leukocytes with the vascular endothelium following an inflammatory stimulus occurs in post-capillary venules; however, leukocytes may also plug capillaries and reduce flow when the microcirculation becomes constricted. As previously described, we also found interaction of leukocytes in pial post-capillary venules after SAH [11,21]. More importantly, we also found capillaries plugged by leukocytes. However, the number of plugged capillaries was quite limited; therefore, leukocyte plugging may not explain the wide-spread reduction in parenchymal perfusion after SAH.

Extravasated perivascular subarachnoid blood, on the other hand, most probably plays an important role in the pathophysiology of MVSs. We demonstrate that this phenomenon mainly occurs in the more superficial vessels (70 µm and less from the brain surface) where MVSs are most pronounced, and this is also where penetrating arterioles originate. Constriction and/or occlusion of penetrating arterioles may thus result in flow disturbance in downstream capillaries and thereby contribute to persistent cerebral hypoperfusion after SAH [22]. Whether the perivascular distribution of blood observed after filament perforation in mice is a common clinical feature after SAH in patients is hard to tell, since clinical imaging modalities do not have the resolution to detect perivascular blood in humans. One study using in vivo imaging, however, showed that patients also have perivascular blood around cortical arterioles after SAH [7]. Hence, we are quite confident that our animal model reflects a clinically relevant situation.

## 4. Materials and Methods

All experimental procedures including the statistical methods and analysis were reviewed and approved by the Animal Ethics Committee of the Government of Upper Bavaria. Sample size was calculated using SigmaPlot 13.0 (Systat Software Inc., Erkrath, Germany). Results are reported according to the ARRIVE guidelines [23]. Given the relevant differences between sexes with respect to stroke pathophysiology and given that this study was designed as a proof-of-principle study with no therapeutic intent, we used only male animals, thereby ensuring comparability of our results with previous studies performed in our laboratory using the same experimental setup. All experiments were performed in a blinded and randomized fashion by drawing lots. Analysis of the data was performed by a researcher unaware of group allocation.

### 4.1. Experimental Groups

**Figure 6:**
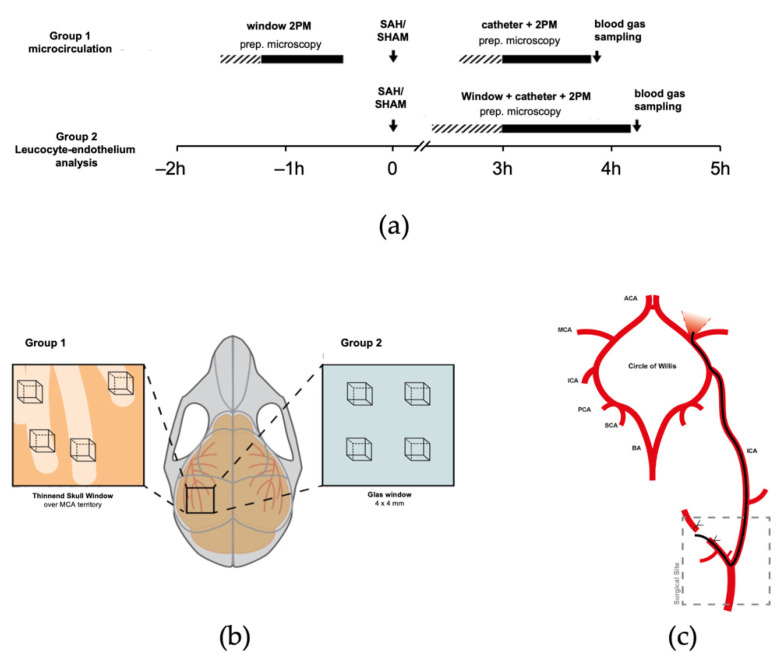
Experimental setup. (**a**) Experimental timeline. B. In vivo two-photon imaging. (**b**) For investigation of microcirculatory perfusion before and after SAH (Group 1), 2-PM was performed via a thinned skull window over the MCA territory (left); for Group 2, leucocyte - endothelium interactions were assessed via an (open) cranial window (right) in four regions of interest. (**c**) The endovascular MCA perforation model of SAH.

In the first set of experiments, we traced blood distribution after SAH (Figure 6a). A closed cranial thinned skull window (Figure 6b, Group 1, *n* = 7 sham, *n* = 7 SAH, 1 SAG drop out) was prepared; after injection of TMRM, the cerebral microcirculation was imaged by 2-PM. Afterwards, SAH or sham surgery was performed; animals were allowed to wake up. Three hours after SAH, anesthesia was re-established, and in vivo microscopy repeated after application of FITC. This setup allowed us to differentiate between blood extravasated during SAH (red fluorescence, TMRM stained) and perfusion after SAH (green fluorescence, FITC staining).

In a second experimental series (Figure 6a, *n* = 7 sham, *n* = 7 SAH, 1 SAG drop out), in vivo microscopy was performed 3 h after SAH via an open cranial window; this technique allows for a higher penetration depth and better image quality, making it more adequate for leucocyte imaging (Figure 6b, Group 2). FITC and rhodamine G were administered as plasma and leukocyte markers, respectively. Sticking leucocytes were traced in pial and parenchymal vessels.

### 4.2. Experimental Subarachnoid Hemorrhage

Sixteen male eight–ten-week-old C57BL/6 mice were subjected to sham surgery (*n* = 8) or experimental subarachnoid hemorrhage (*n* = 8) by endovascular filament perforation under continuous multi-modal physiological monitoring as previously described [9,12,24]. In short, a filament was introduced into the left common carotid artery via the external carotid artery and then advanced towards the circle of Willis (Figure 6c) under continuous control of cerebral blood flow (Laser Doppler Flowmetry, Perimed, Sweden) and intracranial pressure (Codman ICP Express). Successful hemorrhage induction was indicated by a sharp and substantial increase in ICP with a concomitant drop of CBF under 20% of baseline value.

12.5% in the sham surgery group (*n* = 1) and 25% in the SAH group (*n* = 2) dropped out/ were excluded from analysis.

### 4.3. Two-Photon Intravital Microscopy

In vivo two-photon microscopy was performed as previously described [25,26] using a Zeiss LSM 7 microscope equipped with a Li:Ti Laser (Chameleon, Coherent, USA). Fluorescein isothiocyanate (FITC) or tetramethylrhodamine (TMRM) dextran (2,000,000 kD, 0.5% in saline, Sigma Aldrich) was injected as a plasma marker; rhodamine 6G (99%, 0.5% in saline, Sigma Aldrich, St. Louis, MS, USA) was used to label leukocytes. Excitation wavelength was 800 nm for FITC and 840 nm for rhodamine. Fluorescence was detected with GaAsP-NDD detectors (600 digital gain) with a 500–550 nm band-pass filter for FITC and a 565–610 nm band-pass filter for rhodamine. Z-stacks (1 µm step distance) of four different regions of interest (ROI, 600 × 600 µm) were scanned up to 250 µm (thinned skull window) or 500 µm (open window) depth. Blood flow velocity measurements were performed by line scan of a vessel segment 50–100 µm in length; the segment was scanned 1000 times with a minimal pixel dwell time of 0.50–0.64 µs.

### 4.4. Image Analysis

For in vivo experiments, vessel diameter was assessed in vessel segments 60 to 80 µm in length before and after SAH. In total, 89 vessel segments in the sham group and 109 vessel segments in the SAH group were analyzed; in each individual vessel, maximum diameter was determined 5 times. Each ROI was reconstructed in 3D (Imaris 3D, Bitplane) after automated thresholding for correct vessel lumen detection. Total perfused vessel volume was calculated using Imaris. Superficial vessels (0–50 µm below surface), including penetrating arterioles and the parenchymal microcirculation/penetrating vessels, were analyzed separately. Blood flow velocity was determined in line scans by dividing the respective travel time by the velocity of travelling erythrocytes and expressed as µm/ms.

In setup 2, leukocytes were stained with rhodamine 6G. Sticking leukocytes were classified as a red signal larger than 5 µm that did not change position between two scans (10 min apart). In each ROI, sticking leukocytes were counted in superficial and parenchymal (arteriolar, venous) microvessels. 

### 4.5. Statistical Analysis

Sample size calculation and data analysis were performed using SigmaPlot 13 (Systat Software Inc., Erkrath, Germany). Normality testing was undertaken with the Shapiro–Wilk test and, depending on the results, groups were compared using the Mann–Whitney U test or Student’s t-test. Longitudinal ICP and CBF values were compared using ANOVA on ranks for repeated measurements. Differences of *p* ≤ 0.05 were considered to be statistically significant. Data are presented as mean ± standard deviation (SD).

## 5. Conclusions

We show that spasms of pial microvessels following SAH occur more frequently than previously thought, i.e., in almost two thirds of all vessels, and, more importantly, that these spasms cause a 60% reduction in parenchymal perfusion. Plugging of capillaries by leukocytes occurs after SAH but does not seem to add much to the already existing perfusion deficit. Thus, blood-induced spasms of pial arterioles seem to be a major cause of cerebral ischemia after SAH. Deciphering the mechanisms of pial artery spasms may therefore result in novel therapeutic principles for the reduction in cerebral ischemia after SAH.

## Data Availability

All data contained in the present papers are part of Kathrin Nehrkorn’s doctoral thesis. The thesis can be accessed at https://edoc.ub.uni-muenchen.de/19518/ (accessed on 30 July 2021).

## Supplementary Materials

The following are available online at https://www.mdpi.com/article/10.3390/ijms22168444/s1.
