# Supplementary material for: Role of Pial Microvasospasms and Leukocyte Plugging for Parenchymal Perfusion after Subarachnoid Hemorrhage Assessed by In Vivo Multi-Photon Microscopy
Source: Int J Mol Sci. 2021 Aug 6;22(16):8444. doi: 10.3390/ijms22168444 (PMC8395146; doi:10.3390/ijms22168444)
Supplement: Supplementary file 1 [file ijms-22-08444-s001.zip › ijms-1225553-supplementary.pdf]

Supplementary Table S1.

	sham	SAH
**endexpiratory pCO_2 (mmHg)**	23.6 ± 1.6	24.5 ± 1.5
**blood pressure (mmHg)**	86 ± 11	84 ± 16
**temperature (°C)**	37.5 ± 0.1	37.4 ± 0.2
**heart rate (beats/min)**	274 ± 63	306 ± 51
**oxygen saturation (%)**	97.5 ± 1.6	96.8 ± 1.5

Supplementary Table S2.

	sham	SAH
**pH**	7.25 ± 0.15	7.25 ± 0.16
**pCO_2 (mmHg)**	39.6 ± 8.4	39.4 ± 8.1
**pO_2 (mmHg)**	127.1 ± 28.8	118.3 ± 32.1
**Na^+ (mmol/l)**	150 ± 4	151 ± 3
**K^+ (mmol/l)**	4.72 ± 1.02	4.27 ± 0.5
**Ca^{++} (mmol/l)**	1.24 ± 0.12	1.21 ± 0.09
**Htc (%)**	42 ± 4	41 ± 3

Htc hematocrit. SAH subarachnoid hemorrhage.
